# The C-Type Lectin Receptor Mincle Binds to *Streptococcus pneumoniae* but Plays a Limited Role in the Anti-Pneumococcal Innate Immune Response

**DOI:** 10.1371/journal.pone.0117022

**Published:** 2015-02-06

**Authors:** Anne Rabes, Stephanie Zimmermann, Katrin Reppe, Roland Lang, Peter H. Seeberger, Norbert Suttorp, Martin Witzenrath, Bernd Lepenies, Bastian Opitz

**Affiliations:** 1 Department of Internal Medicine/Infectious Diseases and Pulmonary Medicine, Charité Universitätsmedizin Berlin, Berlin, Germany; 2 Max Planck Institute of Colloids and Interfaces, Department of Biomolecular Systems, Potsdam, Germany; 3 Freie Universität Berlin, Institute of Chemistry and Biochemistry, Department of Biology, Chemistry and Pharmacy, Berlin, Germany; 4 Institute for Clinical Microbiology, Immunology and Hygiene, University Hospital Erlangen, Erlangen, Germany; Instituto Butantan, BRAZIL

## Abstract

The innate immune system employs C-type lectin receptors (CLRs) to recognize carbohydrate structures on pathogens and self-antigens. The Macrophage-inducible C-type lectin (Mincle) is a FcRγ-coupled CLR that was shown to bind to mycobacterial cord factor as well as certain fungal species. However, since CLR functions during bacterial infections have not yet been investigated thoroughly, we aimed to examine their function in *Streptococcus pneumonia* infection. Binding studies using a library of recombinantly expressed CLR-Fc fusion proteins indicated a specific, Ca^2+^-dependent, and serotype-specific binding of Mincle to *S. pneumonia*. Subsequent experiments with different Mincle-expressing cells as well as Mincle-deficient mice, however, revealed a limited role of this receptor in bacterial phagocytosis, neutrophil-mediated killing, cytokine production, and antibacterial immune response during pneumonia. Collectively, our results indicate that Mincle is able to recognize *S. pneumonia* but is not required for the anti-pneumococcal innate immune response.

## Introduction

*Streptococcus pneumoniae* frequently colonizes the upper respiratory tract of humans. Depending on the immune status of the host, on preceding viral infections, and on the pneumococcal serotype, this asymptomatic colonization can progress to invasive diseases. These diseases, that include community-acquired pneumonia, sepsis, and meningitis, cause significant mortality especially in children and the elderly [1, 2]. Important virulence factors of *S. pneumoniae* are the exotoxin pneumolysin (PLY) [3], and the polysaccharide capsule that inhibits phagocytosis, complement factor binding, and entrapment by neutrophil extracellular traps [4–6]. The innate immune system detects *S. pneumoniae* through pattern recognition receptors (PRRs) that belong to different protein families and functional classes [7, 8]. For example, the Toll-like receptor (TLR) members TLR2 and TLR9 detect pneumococcal cell wall components and CpG-rich DNA, respectively [9–11]. Among NOD-like receptors (NLRs), NOD2 recognizes pneumococcal peptidoglycan and NLRP3 is activated by PLY [12–15]. Moreover, AIM2 as well as another STING-dependent cytosolic DNA sensor detect pneumococcal nucleic acid in the host cell cytosol [7, 12]. These receptors primarily regulate the production of NF-κB-dependent pro-inflammatory mediators, IL-1 family cytokines, and type I IFNs.

The myeloid C-type lectin receptors (CLRs) represent an additional family of sensors that recognize carbohydrates as well as other ligands of both pathogens and self [16–18]. The CLRs are transmembrane proteins that share a conserved protein fold, termed carbohydrate recognition domain (CRD). The CRD consists of two protein loops and two anti-parallel β-sheets, stabilized by highly conserved disulfide bonds and up to four Ca^2+^-binding sites [19]. Thus, ligand binding by CLRs is often mediated in a Ca^2+^-dependent fashion. The cytoplasmic domains of CLRs frequently contain either hemITAM or ITIM signaling motifs, or associate with ITAM-bearing adaptors such as Fc receptor common γ chain (FcRγ) and DAP12. Whereas hemITAM- and ITAM-mediated signaling stimulates myeloid cell activation through Syk, ITIM-containing CLRs recruit phosphatases and negatively regulate kinase-dependent signaling pathways [16]. While CLRs were shown to interact with a large number of fungi, viruses, or parasites, currently there is limited data available on the function of CLRs in bacterial recognition and the activation of anti-bacterial immune responses [20].

The CLR Macrophage-inducible C-type lectin (Mincle, *Clec4e*) is a type II transmembrane protein that contains a single CRD. Mincle is predominantly expressed by APCs of the myeloid lineage such as macrophages and DCs. The *Mincle* gene is located in the natural killer gene complex together with three related and highly conserved type II CLR genes (encoding MCL, DCIR and Dectin-2), found on murine chromosome 6 (human chromosome 12) [21, 22]. Mincle has been demonstrated to recognize the mycobacterial glycolipid trehalose-6, 6’-dimycolate (TDM, cord factor) [23–25]. Recently, the structural requirements for TDM binding by Mincle have been elucidated by crystallographic analyses [26–28]. In addition, Mincle recognizes *Malassezia* and *Candida* strains, as well as the endogenous ribonucleoprotein SAP130 [29–32]. Since Mincle does not itself express an intracellular signaling domain, it associates with FcRγ chain to stimulate a Syk- and CARD9/Bcl10/Malt1-mediated cascade culminating in the production of NF-κB-dependent proinflammatory cytokines [31, 33]. Fungal engagement of Mincle, however, has also been shown to suppress Dectin-1- and IRF1-mediated IL-12 production by activating the E3 ubiquitin ligase Mdm2 through Syk-CARD9-PI3K [34]. Moreover, Mincle contributes to neutrophil activation, phagocytosis, and bacterial killing upon *M. tuberculosis* and *Klebsiella pneumoniae* infection [35, 36].

In the present study, we used a library of recombinantly expressed CLR-Fc fusion proteins to analyze the contribution of CLRs to *S. pneumoniae* recognition. We identified Mincle as a CLR that bound to *S. pneumoniae* in a Ca^2+^-dependent manner. To analyze whether the Mincle/*S. pneumoniae* interaction impact the immune response, different primary cells and a murine *S. pneumoniae* infection model was employed. However, infection of Mincle- and FcRγ-deficient cells and mice indicated that Mincle did not influence the course of infection suggesting a limited role for Mincle in immunity against *S. pneumoniae*.

## Materials and Methods

### Bacterial strains

*S. pneumoniae* serotype (ST)3 strain PN36 (NCTC7978), ST2 strains D39 and D39Δ*cps*, ST1 multilocus sequence type (MLST)306 strain and ST9N MLST66 strain were used. Bacteria were grown in THY media at 37°C and 5% CO_2_ until they reached a phase of logarithmic growth. For heat-inactivation, bacteria where incubated at 56°C for 1 h while shaking. *Trichosporon cutaneum* was grown in YEPD medium at 26°C for 2–3 days and was then heat-inactivated at 80°C for 20 min.

### Production of the CLR-Fc fusion protein library

The library of CLR-Fc fusion proteins was prepared as described previously [37–39]. Briefly, murine splenic RNA was reverse transcribed into cDNA using Reverse Transcriptase (New England Biolabs, Ipswich, USA). The cDNA encoding the extracellular part of each CLR was amplified by polymerase chain reaction (PCR) and was then ligated into the pFuse-hIgG1-Fc expression vector (InvivoGen, San Diego, USA). The CLR-Fc vector constructs were either stably transfected into CHO cells or transiently transfected using the FreeStyle Max CHO-S Expression System (Life Technologies, Darmstadt, Germany). Purification of the CLR-Fc fusion proteins from the cell supernatant was performed using HiTrap Protein G HP columns (GE Healthcare, Piscataway, USA). The purity of each CLR-Fc fusion protein was confirmed by sodium dodecyl sulfate polyacrylamide gel electrophoresis (SDS-PAGE) and subsequent Coomassie stain, Western Blot using anti-human IgG-HRP antibody (Dianova, Hamburg, Germany) as well as mass spectrometry.

### Binding of Mincle-Fc to immobilized *S. pneumoniae*

Heat inactivated bacteria diluted in PBS were coated on 96-well high binding plates (Greiner, Frickenhausen, Germany) at a concentration of 3×10^8^ cells/mL overnight. After blocking with 1% BSA in PBS, 10 μg/mL of each CLR-Fc fusion protein was incubated in lectin binding buffer (50 mM HEPES, 5 mM MgCl_2_, 5 mM CaCl_2_, pH 7.4) or EDTA buffer (10 mM EDTA, 50 mM HEPES, pH 7.4) at RT for 2 h. Binding of Mincle to plate-bound trehalose-6, 6’-dibehenate (TDB, 50 µg/mL) was used as positive control. For competition experiments, heat-inactivated bacteria were pre-incubated with 10 µg/mL Mincle-Fc at 4°C for 15 min. The binding of CLR-Fc fusion proteins was detected by an alkaline phosphatase-conjugated goat anti-hFc antibody (Dianova, Hamburg, Germany). Development was performed with *p*-nitrophenyl phosphate (Thermo Scientific, Rockford, IL, USA).

### Flow cytometric analysis of Mincle-Fc binding to *S. pneumoniae*

Heat-inactivated bacteria were labeled with SYTO61 Red Fluorescent Nucleic Acid Stain (2.5 µM in PBS; Life Technologies) at RT for 30 min and were afterwards washed three times in PBS. To analyze the Ca^2+^ dependency of the interaction, 50 µL labeled bacteria (3×10^8^ cells/mL) were incubated with 20 µg/mL Mincle-Fc diluted in lectin binding buffer or EDTA buffer at 4°C for 1 h. After three washing steps with PBS, Mincle-Fc binding to *S. pneumoniae* was detected with a PE-conjugated goat anti-hFc antibody (Dianova, Hamburg, Germany). Flow cytometric analysis was performed with a FACSCanto II flow cytometer (BD Pharmingen, Heidelberg, Germany). Data were analyzed using the FlowJo analysis software (Tree Star Inc., Ashland, OR, USA).

### Cells and infection

Primary cells were isolated from wild-type (WT), *Fcerg1^-/-^* (encoding FcRγ) [40] or *Mincle^-/-^* [29] mice on a C57Bl/6J background. Alveolar macrophages (AMΦs) were obtained by bronchoalveolar lavage from mouse lungs. For isolation of alveolar epithelial cells (AECs), lung homogenates were prepared and leukocytes and endothelial cells were depleted from the cell suspension by incubation with biotinylated rat anti-mouse CD45, CD16/32 and CD31 (BD Pharmingen, Heidelberg, Germany) followed by magnetic separation. Microvascular endothelial cells (MVECs) were isolated from lung homogenates by positive magnetic selection using biotinylated rat anti-mouse CD144 (BD Pharmingen). For culturing, plates were coated with fibronectin. Bone marrow-derived macrophages (BMMs) were prepared from the bone marrow and cultured in RPMI 1640 containing 30% L929 cell supernatant and 20% FCS for 10 days. Polymorphonuclear leukocytes (PMNs) were isolated from the bone marrow using the anti-Ly6G MicroBead Kit (Miltenyi Biotech, Bergisch Gladbach, Germany). Cells were infected with *S. pneumoniae* ST2 (D39) or were stimulated with TDM (Sigma, St. Louis, USA) or LPS (Alexis Biochemicals, San Diego, USA).

### Bacterial uptake and killing assays

BMMs were infected with *S. pneumoniae* ST2 (D39Δ*cps*) for 30 min followed by treatment with 50 mg/mL gentamicin. Intracellular, viable bacteria were determined 30 min or 90 min after treatment. Cells were PBS-washed and lysed with 1% saponin for 10 min. Serial dilutions of the bacterial suspensions were plated on blood agar plates, and CFUs were determined. In PMNs, opsonophagocytic killing assays were conducted as described previously [41]. In brief, *S. pneumoniae* ST2 (D39Δ*cps*) were pre-opsonized with infant rabbit serum (Pel-Freez Biologicals, Rogers, USA) at 37°C for 30 min before PMN were added. The percentage of viable bacteria was determined relative to control reactions lacking neutrophils after incubation at 37°C for 45 min.

### Ethics statement

Animal experiments were performed in strict accordance with the German regulations of the Society for Laboratory Animal Science and the European Health Law of the Federation of Laboratory Animal Science Associations. The protocol was approved by the Landesamt für Gesundheit und Soziales Berlin (Permit No. G 0210/11, G 0304/12, G 0357/12). All efforts were made to minimize suffering.

### Murine pneumonia model

Female 8–10 weeks old WT, *Fcer1g^-/-^* and *Mincle^-/-^* mice on C57Bl/6J background were housed in environmentally enriched and individually ventilated cages under specific pathogen free conditions. Food and water was given ad libitum. For infection, mice were anesthetized by i.p. ketamine (80 mg/kg) and xylazine (25 mg/kg) and transnasally inoculated with 7.5×10^4^ or 5×10^6^ CFU *S. pneumoniae* ST3 (PN36) in 20 μL PBS or sham-infected with 20 µL PBS as described previously [13]. Mice were monitored every 12 h (more often if severely ill) and were humanely sacrificed by i.p. ketamine (240 mg/kg) and xylazine (112, 5 mg/kg) when they reached the predefined humane endpoints (body temperature < 30°C, body weight loss > 20%). Survival was recorded for 10 days or mice were sacrificed 6, 12, 24, or 48 h p.i. For analysis, anesthetized (160 mg/kg mg ketamine, 75 mg/kg xylazine) mice were heparinized, tracheotomised and ventilated and perfused via the pulmonary artery with saline for 2 min.

### Determination of bacterial load, cell recruitment and cytokines

Bacterial loads were determined in the bronchoalveolar lavage fluid (BALF) and blood. Serial dilutions of samples were plated on blood agar and CFUs were determined. BAL cells were counted by haemocytometer and differentiated by flow cytometry (FACSCalibur; BD) as described previously [13]. Cytokines were quantified by ELISA in the BALF or by quantitative RT-PCR from total cellular RNA of the lung.

### ELISA

Concentrations of IL-6, TNFα and KC in the BAL or in cell-free supernatants were quantified by commercially available sandwich ELISA kits (eBioscience, Frankfurt, Germany; R&D, Minneapolis, USA).

### Quantitative RT-PCR analysis

Total cellular RNA was isolated, transcribed to cDNA, and amplified by quantitative RT-PCR using Gene Expression Master Mix (Applied Biosystems, Foster City, USA). TaqMan Gene Expression Assays were purchased from Applied Biosystems.

### Data Analysis

Data are expressed as mean ± SEM. For CLR-Fc binding studies, statistical analysis was performed using unpaired Student’s t-test. For murine *S. pneumoniae* infection experiments, analysis was performed using the log-rank test for survival, and the Kruskal–Wallis test followed by Dunn’s multiple comparison test for comparison of more than two groups. Data analysis was performed using the Prism software (GraphPad Software, La Jolla, CA). For all statistical analyses, p values < 0.05 were considered significant: *p < 0.05, **p < 0.01, ***p < 0.001, ****p < 0.0001.

## Results

### Mincle binds to *S. pneumoniae*

**Figure 1:**
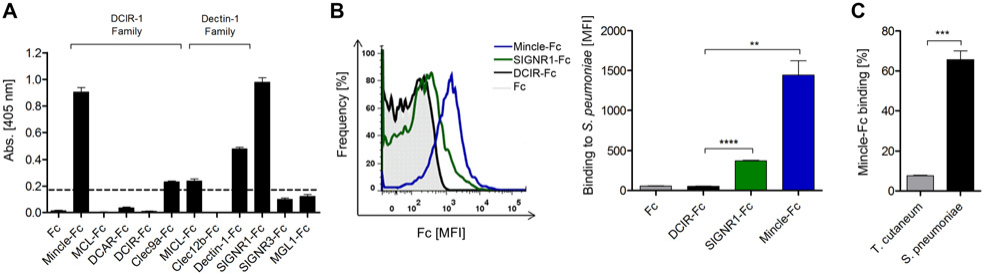
Mincle binds to *S. pneumoniae*. (A) Heat-inactivated *S. pneumoniae* ST3 (1.5×10^7^ cells/well) diluted in PBS were immobilized on ELISA plates and incubated with CLR-Fc fusion proteins (10 µg/mL) diluted in lectin binding buffer. Data are representative of three independent experiments (triplicates each). The dashed line indicates background binding of CLR-Fc fusion proteins. (B) Binding of Mincle-Fc, SIGNR1-Fc and DCIR-Fc was analyzed by flow cytometry. CLR-Fc fusion proteins (20 µg/mL) were incubated with *S. pneumoniae* ST3 at a concentration of 3×10^8^ cells/mL diluted in lectin binding buffer. Left: Representative histogram plot of one binding experiment. Right: Statistical analysis of the flow cytometry-based binding assay. Data are representative of three independent experiments (triplicates each). (C) Mincle-Fc binding to heat-killed *T. cutaneum* and *S. pneumoniae* ST3 was analyzed by flow cytometry. Percentage of binding is shown relative to Fc binding to each pathogen. Data are representative of three independent experiments (triplicates each). (A-C), Data are shown as mean + SEM. Significance is indicated by asterisks, * = p<0.05; ** = p<0.01; *** = p<0.001; **** = p<0.0001.

To analyze whether CLRs are involved in *S. pneumoniae* recognition, we performed an initial ELISA screening for CLR binding to heat-killed *S. pneumoniae* ST3 using a comprehensive library of CLR-Fc fusion proteins [37] (Fig. 1A). Binding of the murine DC-SIGN homolog Specific intercellular adhesion molecule-grabbing nonintegrin receptor 1 (SIGNR1) was used as a positive control since SIGNR1 on marginal zone macrophages was previously reported to be crucial for *S. pneumoniae* recognition [42]. Indeed, SIGNR1-Fc exhibited substantial binding to plate-bound *S. pneumoniae* (Fig. 1A). Besides SIGNR1-Fc, the ELISA-based pre-screening revealed Mincle as a candidate CLR that bound to heat-killed *S. pneumoniae* (Fig. 1A). In contrast, numerous other CLR-Fc fusion proteins, including MCL, DCAR, DCIR, CLEC-9a, MICL, CLEC-12b, SIGNR3, and MGL1 exhibited no or very weak binding to plate-bound *S. pneumoniae*. To confirm the specificity of the Mincle/*S. pneumoniae* ST3 interaction, we performed flow cytometric binding assays (Fig. 1B–C). In agreement with the ELISA-based binding assay, flow cytometric analysis indicated substantial binding of Mincle-Fc to *S. pneumoniae* ST3 (Fig. 1B). Furthermore, we performed comparative binding studies with Mincle-Fc and other bacteria and fungi. In a previous study, the fungus *T. cutaneum* did not induce Mincle activation in a Mincle reporter cell line-based assay [32]. Indeed, whereas a high percentage of *S. pneumoniae* was recognized by Mincle-Fc, we observed only marginal binding of Mincle-Fc to *T. cutaneum*. These findings indicate that Mincle specifically recognizes *S. pneumoniae*.

### The Mincle/*S. pneumoniae* interaction is Ca^2+^-dependent and serotype specific

**Figure 2:**
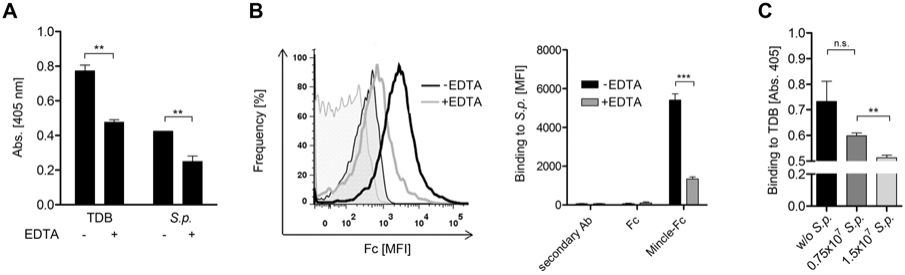
Mincle binding to *S. pneumoniae* is Ca^2+^-dependent. (A) To analyze the Ca^2+^ dependency of the Mincle/*S. pneumoniae* interaction, TDB (50 µg/mL in isopropanol) and heat-inactivated *S. pneumoniae* ST3 (3×10^8^ cells/mL in PBS) were immobilized on ELISA plates. Binding of Mincle-Fc (10 µg/mL) diluted in lectin binding buffer or EDTA buffer (10 mM EDTA) to immobilized TDB and *S. pneumoniae* ST3 was analyzed. Data are representative of three independent experiments (triplicates each). (B) Mincle-Fc (20 µg/mL) (non-filled curves) or Fc only (gray-filled curves) were incubated with *S. pneumoniae* ST3 (3×10^8^ cells/mL) in lectin binding buffer or EDTA buffer (10 mM EDTA). Left: Representative histogram plot of one binding experiment. Right: Statistical analysis of the flow cytometry-based binding assay in the presence or absence of EDTA. Data are representative of three independent experiments (triplicates each). (C) 20 µg/mL Mincle-Fc, diluted in lectin binding buffer, was pre-incubated with *S. pneumoniae* ST3 and subsequently incubated with plate-bound TDB (50 µg/mL). Data are representative of three independent experiments (triplicates each). (A-C), Data are shown as mean + SEM. Significance is indicated by asterisks, * = p<0.05; ** = p<0.01; *** = p<0.001.

Next, we determined whether the Mincle-Fc binding to *S. pneumoniae* was mediated in a Ca^2+^-dependent manner. Previously, it was shown that Mincle binds to its known ligands TDM (cord factor) and TDB in a Ca^2+^ dependent fashion [24, 25]. Pre-incubation of Mincle-Fc in a buffer containing the Ca^2+^ chelating agent EDTA resulted in reduced binding of Mincle-Fc to plate-bound TDB as well as *S. pneumoniae* ST3 suggesting a Ca^2+^-dependent Mincle/*S. pneumoniae* interaction (Fig. 2A). This finding was corroborated by flow cytometry since the Mincle-Fc incubation in an EDTA-containing buffer markedly reduced its binding to *S. pneumoniae* (Fig. 2B). Next, we performed an ELISA-based competition assay with increasing concentrations of heat-killed *S. pneumoniae* ST3 to disrupt the Mincle-Fc binding to the Mincle ligand TDB (Fig. 2C). Indeed, Mincle-Fc incubation in the presence of *S. pneumoniae* led to reduced binding to TDB, thus confirming the specificity of the Mincle/*S. pneumoniae* interaction. To analyze if Mincle binding to *S. pneumoniae* is serotype specific, we performed binding studies with different *S. pneumoniae* serotypes (ST1, ST2, ST3 and ST9N). Indeed, we observed a differential binding of Mincle-Fc to different serotypes. Mincle-Fc exhibited a strong binding to *S. pneumoniae* ST2 (D39) and ST3 (PN36), weaker binding to ST9N and almost no binding to ST1 (S1 Fig.). Thus, we conclude that Mincle-Fc binding to *S. pneumoniae* is serotype-specific suggesting a carbohydrate-specific binding of Mincle to *S. pneumoniae*.

### Mincle is not required for production of inflammatory cytokines, phagocytosis or bacterial killing upon *S. pneumoniae* infection

**Figure 3:**
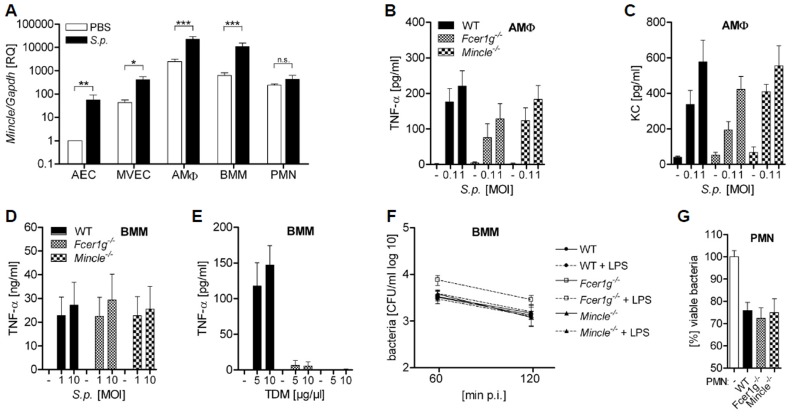
Lack of FcRγ or Mincle does not affect the innate immune response to *S. pneumoniae* in different cell types. (A) AECs, MVECs and AMΦs were isolated from the lung, and PMNs and BMMs from the bone marrow of C57Bl/6 mice. Cells were left untreated or infected with *S. pneumoniae* ST2 (D39). (B-G) AMΦs, BMMs and PMNs were isolated from WT, *Fcer1g^-/-^* and *Mincle^-/-^* mice. (B, C) AMΦs or (D) BMMs were infected with *S. pneumoniae* ST2 or (E) BMMs were stimulated with TDM as a positive control for 16 h and cytokine release was quantified by ELISA. (F) Untreated or LPS-treated (100 ng/ml, 4 h) BMMs were infected with *S. pneumoniae* ST2Δ*cps* (MOI = 2.5) and were treated with gentamicin (50 mg/ml) after 30 min. Intracellular, viable bacteria were determined 60 min or 120 min post infection. (G) Rabbit serum-opsonized *S. pneumoniae* ST2Δ*cps* were incubated with PMNs and neutrophil-mediated killing was assessed after 45 min incubation. Data are shown as mean + SEM of (A) two (A: PMN, E, F), three (C, G), four (A: AEC, MVEC, B, D) or five (A: AMΦ, BMM) independent experiments carried out in duplicates (A-F) or quadruplicates (G); * = p<0.05; ** = p<0.01; *** = p<0.001; n.s. not significant.

Next, we examined the expression and function of Mincle in different cell types. We found that Mincle expression is low in alveolar epithelial cells and lung microvascular endothelial cells, and higher in alveolar macrophages, bone-marrow macrophages (BMMs) and neutrophils (Fig. 3A). Mincle expression was up-regulated upon pneumococcal infection in lung epithelial cells, endothelial cells and macrophages. Lack of Mincle or FcRγ (*Fcerg1-/-)* in alveolar macrophages and BMMs did not significantly affect the *S. pneumoniae* ST2-induced production of TNFα or KC (Fig. 3B–D). In contrast, cytokine production stimulated by TDM, which served as a positive control, was abolished in *Mincle^-/-^* and *Fcer1g^-/-^* cells cells (Fig. 3E). Moreover, phagocytosis and killing of *S. pneumoniae* by macrophages and neutrophils were not significantly affected by Mincle or FcRγ deficiency (Fig. 3F–G). Thus, our data suggest that *S. pneumoniae* recognition by Mincle and Mincle-dependent signaling might be dispensable for the innate immune response of macrophages and neutrophils towards *S. pneumoniae*.

### Mincle is not required for the antibacterial innate immune response during pneumococcal pneumonia

**Figure 4:**
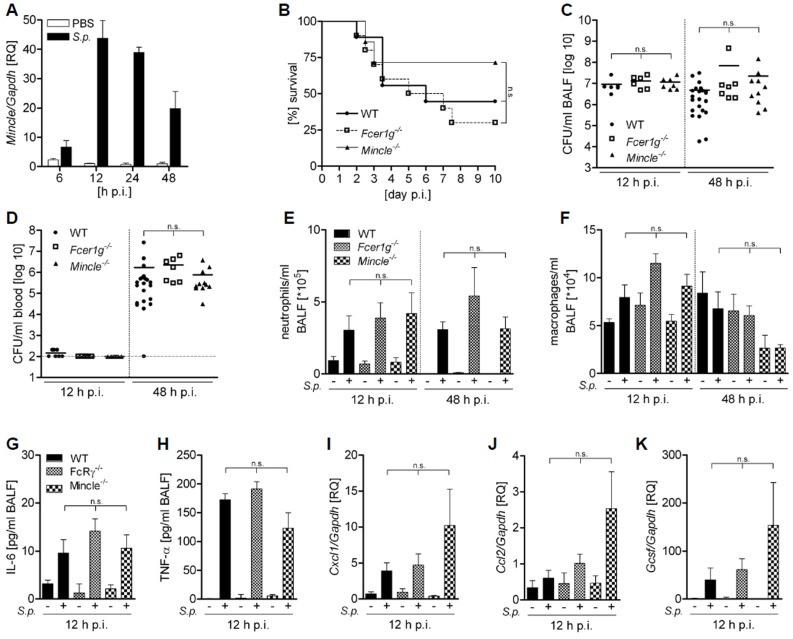
Lack of Mincle or FcRγ does not influence the antibacterial host response during pneumococcal pneumonia. (A) C57Bl/6 mice were intranasally infected with 5×10^6^ CFU/mouse *S. pneumoniae* ST3 or control treated with PBS and Mincle expression levels in the lungs at the indicated time points were determined by quantitative RT-PCR. (B) WT, *Fcerg1^-/-^* and *Mincle^-/-^* mice were intranasally infected with 7.5×10^4^ CFU/mouse *S. pneumoniae* ST3 and survival was monitored every 12 h over 10 days. (C-K) WT, *Fcer1g^-/-^* and *Mincle^-/-^* mice were intranasally infected with 5×10^6^ CFU/mouse *S. pneumoniae* ST3 or treated with PBS. Bacterial loads were determined in the (C) bronchoalveolar lavage fluid (BALF) and (D) blood. (E) Neutrophils and (F) macrophages in the BALF were quantified by flow cytometry. (G) IL-6 and (H) TNFα (H) levels in the BALF were analyzed by ELISA. Relative expression of (I) *Cxcl1*, (J) *Ccl2* and (K) *Gcsf* in the lung was determined by quantitative RT-PCR. Data are shown as mean + SEM; (A) n = 3, (B) n = 8–10, (C-F) n = 6–22, (G-H) n = 6–8, (I-K) n = 4–5 mice each group. n.s. not significant.

Although Mincle signaling did not affect innate immune responses of different cell subsets to *S. pneumoniae in vitro*, a Mincle-dependent bacterial detection in other cell types, or alternatively Mincle-mediated responses to endogenous danger molecules such as SAP130 [31] could regulate the antibacterial immune response *in vivo*. We therefore examined the function of Mincle and its adapter molecule FcRγ during pneumococcal pneumonia. Intranasal infection of wild-type mice with *S. pneumoniae* ST3 led to increased expression of Mincle in the whole lung (Fig. 4A). However, *Mincle^-/-^, Fcer1g^-/-^* and wild-type mice did not significantly differ in their survival following *S. pneumoniae* infection (Fig. 4B). Moreover, mice lacking Mincle or its adapter molecule displayed unaltered bacterial loads in the bronchoalveolar lavage fluid (BALF) as compared to wild-type animals (Fig. 4C). To evaluate systemic dissemination of *S. pneumoniae* in these animals, bacterial loads in blood and spleen were determined. We detected high bacterial numbers in blood and spleens of all groups of infected mice 48 h p.i. (Fig. 4D and data not shown). According to the unaltered bacterial loads, *S. pneumoniae*-infected *Mincle^-/-^, Fcer1g^-/-^* and wild-type mice exhibited a similar recruitment of neutrophils and macrophages to the lung (Fig. 4E–F). In addition, *S. pneumoniae*-induced production of cytokines and chemokines, many of which have been previously associated with Mincle activation in different models [24, 31], were not significantly affected by lack of Mincle or FcRγ (Fig. 4G–K). These data collectively indicate that Mincle signaling is not required for antibacterial innate immune responses and resistance to *S. pneumoniae* ST3 *in vivo*.

## Discussion

Myeloid CLRs are an important class of PRRs expressed by various immune cells and capable of detecting a broad spectrum of microbial and endogenous ligands. However, their function in acute bacterial infection has not been investigated systematically. For *S. pneumoniae* infection, SIGNR1 is one of the few CLRs shown to be crucial for bacterial recognition, phagocytosis as well as antibacterial defense [43, 44]. We therefore set out to identify additional CLRs that are able to bind to *S. pneumoniae*. To this end, we used a comprehensive library of CLR-Fc fusion proteins covering immunologically relevant members of the Dectin-1 and DCIR family [37]. CLR-Fc fusion proteins are useful tools to identify pathogen/CLR interactions as they display the CRD in a dimeric fashion. Dimeric display allows for multivalent ligand binding and has helped to unravel novel CLR interactions with microbes or endogenous ligands [45, 46]. Out of our CLR-Fc library, we identified Mincle as a novel binder of *S. pneumoniae*, in addition to the known pneumococcal recognition by SIGNR1. We demonstrate that the Mincle/*S. pneumoniae* interaction occurs in a Ca^2+^-dependent fashion, as has been previously shown for the Mincle ligand TDM and pathogenic fungi [24, 30, 32].

To determine whether the Mincle/*S. pneumoniae* interaction impacts the innate immune response, we employed various primary immune cells and the murine infection model of pneumococcal pneumonia. However, we did not observe a functional consequence of this interaction for *S. pneumoniae*-induced innate responses *in vitro* and *in vivo*. Our finding that Mincle and FcRγ-deficiencies did not affect cytokine production and phagocytosis in macrophages suggests that binding of *S. pneumoniae* to Mincle does not correlate with down-stream signaling. Alternatively, the loss of Mincle signaling in macrophages, neutrophils and mice might have been compensated by other functionally redundant CLRs not coupled to FcRγ, as commonly observed in different infection models using CLR-deficient cell and mouse lines [16]. In addition, PRRs such as the TLRs and NLRs known to be essential for antibacterial immunity against *S. pneumoniae* [2, 47] might have overcome potential Mincle-mediated effects. It appears reasonable that the recognition of prokaryote-specific PAMPs such as peptidoglycan and pore-forming toxins by those receptors might dominate the innate immune response to bacteria. In contrast, the recognition of broadly expressed carbohydrate ligands might play a more important and non-redundant role in infections with eukaryotic pathogens such as fungi and parasites [20, 48].

Mincle is known to recognize different glycolipids in *M. tuberculosis* and fungi [24, 27, 30]. Given that glycolipids such as lipoteichoic acid are also present in *S. pneumoniae* [49, 50], it is reasonable to speculate that those structures mediate the binding to Mincle. In addition, Mincle also binds to the endogenous protein SAP130, indicating that besides glycolipids non-glycosylated ligands may be recognized by Mincle as well [31]. This renders the recognition of a protein ligand present in *S. pneumoniae* possible, although it appears unlikely since all binding studies were performed using pneumococci after heat inactivation. The specificity of the *S. pneumoniae*/Mincle interaction was proven by different observations. First, we detected no or only marginal binding of most other CLR-Fc fusion proteins of the library to the bacterium, second the interaction was Ca^2+^-dependent, and third, it could be competitively inhibited. Considering this specific interaction, it may be interesting to elucidate in future studies whether combined deficiencies in Mincle and other CLRs and/or cross-talk mechanisms between Mincle and other PRRs act in a synergistic manner.

## Supporting Information

S1 Fig Binding of Mincle to *S. pneumoniae* is serotype-specific. Binding of Mincle-Fc to different *S. pneumoniae* serotypes was analyzed by flow cytometry. Mincle-Fc and hFc (20 μg/mL) were incubated with *S. pneumoniae* serotype 1, serotype 2 (D39), serotype 3 (PN36), and serotype 9N at a concentration of 3×10^8^ cells/mL in lectin binding buffer. Bound fusion protein was detected by a PE-conjugated goat anti-hFc antibody. Results are shown as MFI values (mean + SEM) and are representative of three independent experiments (triplicates each). Significance is indicated by asterisks, *=p<0.05; **=p<0.01; ***=p<0.001; ****=p<0.0001; ns = non-significant.
(TIF)
