# Supplementary figures and images for: The C-Type Lectin Receptor Mincle Binds to Streptococcus pneumoniae but Plays a Limited Role in the Anti-Pneumococcal Innate Immune Response
Source: PLoS One. 2015 Feb 6;10(2):e0117022. doi: 10.1371/journal.pone.0117022 (PMC4319728; doi:10.1371/journal.pone.0117022)

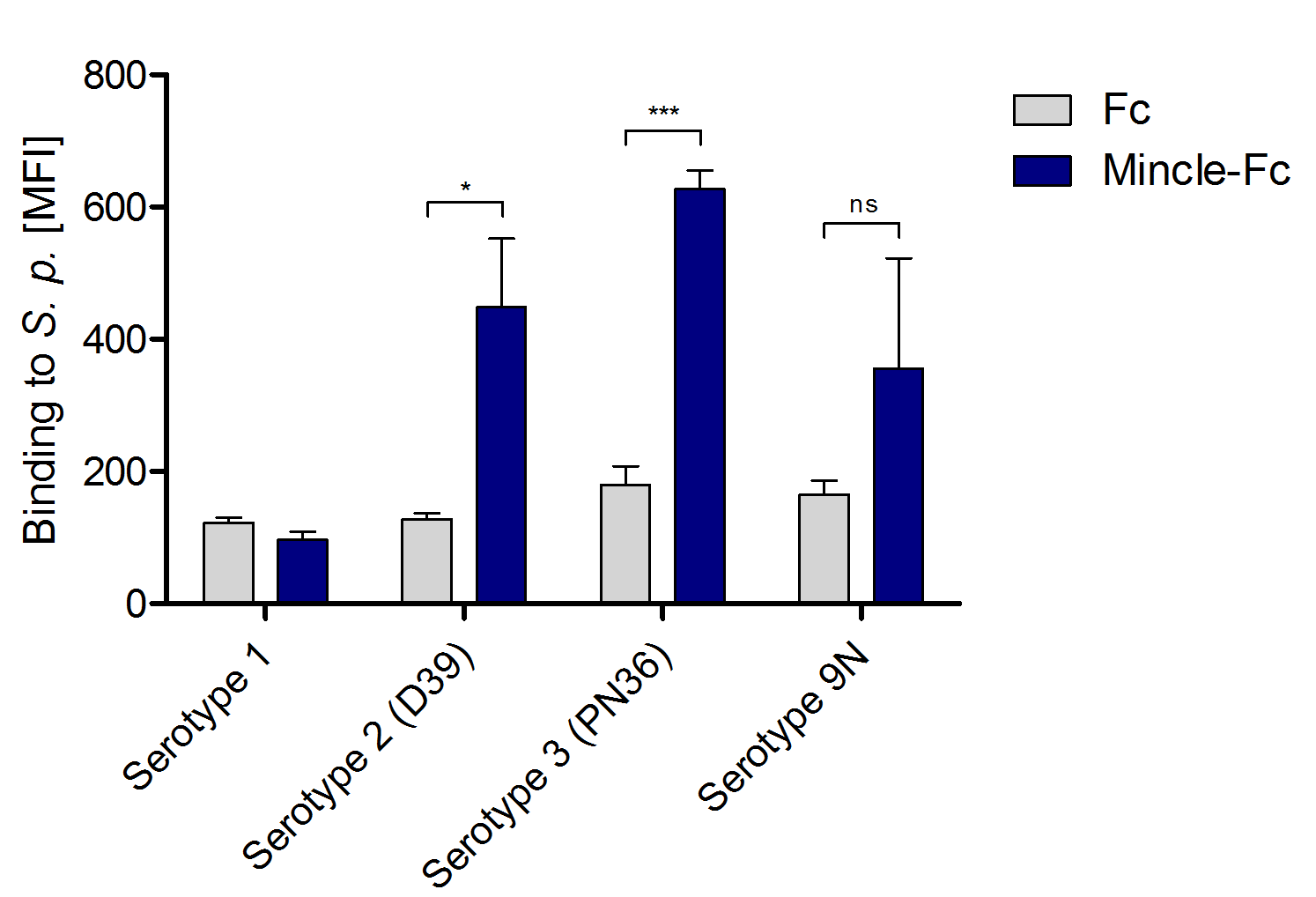

Supplement: S1 Fig — Binding of Mincle-Fc to different S. pneumoniae serotypes was analyzed by flow cytometry. Mincle-Fc and hFc (20 μg/mL) were incubated with S. pneumoniae serotype 1, serotype 2 (D39), serotype 3 (PN36), and serotype 9N at a concentration of 3×10^8^ cells/mL in lectin binding buffer. Bound fusion protein was detected by a PE-conjugated goat anti-hFc antibody. Results are shown as MFI values (mean + SEM) and are representative of three independent experiments (triplicates each). Significance is indicated by asterisks, *=p<0.05; **=p<0.01; ***=p<0.001; ****=p<0.0001; ns = non-significant. (TIF) [file pone.0117022.s001.tif]
